# Substitution of Fish for Red Meat or Poultry and Risk of Ischemic Stroke

**DOI:** 10.3390/nu10111648

**Published:** 2018-11-03

**Authors:** Stine K. Venø, Christian S. Bork, Marianne U. Jakobsen, Søren Lundbye-Christensen, Flemming W. Bach, Peter L. McLennan, Anne Tjønneland, Erik B. Schmidt, Kim Overvad

**Affiliations:** 1 Department of Cardiology, Aalborg University Hospital, 9000 Aalborg, Denmark; c.bork@rn.dk (C.S.B.); ebs@rn.dk (E.B.S.); ko@ph.au.dk (K.O.); 2 Department of Clinical Medicine, Aalborg University, 9000 Aalborg, Denmark; 3 National Food Institute, Division for Diet, Disease Prevention and Toxicology, Technical University of Denmark, 2800 Kgs. Lyngby, Denmark; muja@food.dtu.dk; 4 Unit of Clinical Biostatistics, Aalborg University Hospital, 9000 Aalborg, Denmark; solc@rn.dk; 5 Atrial Fibrillation Study Group, Aalborg University Hospital, 9000 Aalborg, Denmark; 6 Department of Neurology, Aarhus University Hospital, DK-8000 Aarhus C, Denmark; flemming.bach@rm.dk; 7 School of Medicine, University of Wollongong, Wollongong, NSW 2522, Australia; petermcl@uow.edu.au; 8 Danish Cancer Society Research Center, DK-2100 Copenhagen Ø, Denmark; annet@CANCER.DK; 9 Department of Public Health, University of Copenhagen, 2200 Copenhagen N, Denmark; 10 Department of Public Health, Aarhus University, DK-8000 Aarhus C, Denmark

**Keywords:** ischemic stroke, TOAST classification, substitution models, cohort studies, diet, fish, meat, poultry

## Abstract

We investigated the risk of ischemic stroke and its subtypes when red meat or poultry was substituted with fish. A total of 57,053 participants aged 50–65 years at baseline were included in the Danish Diet, Cancer and Health study. All participants filled in a food-frequency questionnaire at recruitment. Potential ischemic stroke cases were identified by linkage to the Danish National Patient Register, and all cases were validated and subclassified. Substitutions were investigated as 150 g/week of fish for 150 g/week of red meat or of poultry using multivariable Cox proportional hazard regression models. During 13.5 years of follow-up, 1879 participants developed an ischemic stroke. Replacing red meat or poultry with fish was not associated with the rate of total ischemic stroke, but there was a statistically significant lower rate of large artery atherosclerosis when fish replaced processed (hazard ratio (HR): 0.78; 95% confidence interval (CI): 0.67; 0.90) and unprocessed (HR: 0.87; 95% CI: 0.75; 0.99) red meat. A statistically significant higher rate of cardioembolism was found when poultry was replaced by total fish (HR: 1.42; 95% CI: 1.04; 1.93). When fatty fish replaced unprocessed red meat, a statistically significant lower rate of small-vessel occlusion was found (HR: 0.88; 95% CI: 0.77; 0.99). In conclusion, replacing red meat with fish was not associated with risk of total ischemic stroke but was associated with a lower risk of subtypes of ischemic stroke.

## 1. Introduction

Ischemic stroke is a leading cause of death and disability worldwide [1]. Because of the often irreversible and devastating consequences of ischemic stroke, prevention is essential. The health benefits of fish intake have been of interest for several decades and some studies have shown beneficial associations between a high fish consumption and ischemic stroke risk [2,3,4]. However, most studies evaluating fish intake in relation to ischemic stroke risk have, assuming energy balance, not taken into consideration that a higher fish intake must necessarily be accompanied by a concomitant lower intake of other foods [5]. Since the food being replaced may attenuate or augment a beneficial effect of fish consumption, the association between fish intake and ischemic stroke risk also depends on the replaced foods. Fish contains different important nutrients, and importantly marine *n*-3 polyunsaturated fatty acids (PUFA), which have shown beneficial effects on risk factors of ischemic stroke including blood pressure, plasma triglycerides, and measures of inflammation [6,7,8].

The most obvious food items that fish would replace are red meat and poultry. Though results have been inconsistent, intake of red meat, especially processed red meat, has been associated with a higher risk of ischemic stroke [9,10,11,12,13,14], while studies of poultry intake have shown no associations with risk of ischemic stroke [11,12,13].

In this study we aimed to investigate the risk of ischemic stroke and its subtypes when processed or unprocessed red meat or poultry were replaced with fish (total, lean, or fatty).

## 2. Materials and Methods

### 2.1. Study Population

The Diet, Cancer and Health cohort study was initiated between 1993 and 1997, when 160,725 persons were invited to participate [15]. Eligible participants were aged between 50–64 years, living in and around Copenhagen and Aarhus in Denmark, and had no previous cancer diagnosis. At baseline, participants gave written informed consent and filled in a validated 192-item semiquantitative food frequency questionnaire [16,17] and a lifestyle questionnaire, and underwent a physical examination. The study was approved by the local Ethics committees and the Danish Data Protection Agency. Participants were excluded if they had cancer or stroke before recruitment. We subsequently also excluded participants for whom information on exposures of interest, or covariates was missing. 

### 2.2. Exposure

The intake of foods and alcohol was assessed from the food frequency questionnaire and was reported as the average intake during the previous year. Consumption of food items were reported in 12 categories, ranging from never to more than eight times/day. A total of 24 questions covered the intake of fish, while intakes of red meat and poultry were covered by 35 and 4 questions, respectively. Calculation of the daily intake of food items was done using the software program FoodCalc (http://www.ibt.ku.dk/jesper/foodcalc). Exposures in this study were intake of total, lean, and fatty fish, processed and unprocessed red meat, and poultry. Lean fish was defined by a low content of n-3 PUFA (≤1 g/100 g) while fatty fish was defined by a high content of *n*-3 PUFA (>1 g/100 g). Processed red meat included red meat that had undergone smoking, salting or curing. Exposures were investigated as substitutions of 150 g/week, corresponding to a common serving size.

### 2.3. Covariates

In the lifestyle questionnaire participants gave information on education, physical activity and smoking status. Hypercholesterolemia, hypertension and diabetes mellitus were self-reported and/or defined by the use of medication for the respective conditions. Information on atrial fibrillation/atrial flutter was obtained by linkage with the Danish National Patient Register using the International Classification of Diseases (ICD)-8 discharge codes 42793 or 42794 and ICD-10 discharge code I489.

### 2.4. Ischemic Stroke Cases

The outcome measure was incident ischemic stroke as well as subtypes of ischemic stroke. Stroke was defined as an acute disturbance of focal or global cerebral function with symptoms lasting more than 24 h. By linkage to the Danish National Patient Register possible stroke cases within the cohort were identified according to ICD-8 discharge codes 430, 431, 433, 434, 436.01, or 436.90, or ICD-10 discharge codes I60, I61, I63 or I64. Medical records and hospital discharge letters were reviewed, and diagnoses validated and characterized on the basis of clinical appearance, computed tomography, magnetic resonance imaging scan, autopsy records, spinal fluid examination and other relevant information [18,19]. All ischemic stroke cases were subclassified according to The Trial of Org 10172 in Acute Stroke Treatment (TOAST) classification [20] and the subtypes included large artery atherosclerosis, cardioembolism, small-vessel occlusion, stroke of other etiology, and stroke of undetermined etiology. Participants were followed from the date of enrolment until occurrence of ischemic stroke, death from another cause, emigration, or end of follow-up (December 2009).

### 2.5. Statistical Analyses

We used sex-stratified Cox proportional hazard regression models allowing for different baseline hazards between men and women, to calculate hazard ratios (HR) for total ischemic stroke and for ischemic stroke subtypes with 95% confidence intervals (CI). Age was used as the underlying time axis. By using statistical food substitution models, we investigated the rate of ischemic stroke and its subtypes when intake of 150 g/week of total, lean or fatty fish replaced processed or unprocessed red meat or poultry. 

Adjustment for potential confounders and risk factors was undertaken in three steps. In Model 1A, we adjusted for age at baseline (years, continuous), sex, and energy intake (kcal/week). In Model 1B we further adjusted for length of schooling (<7 years, 8–10 years, >10 years), waist circumference adjusted for Body mass index (cm, continuous), smoking status (non-current smoker, current smoker <15, or ≥15 g tobacco/day), physical activity (hours/week, continuous), alcohol intake (g/d, continuous) and abstinence from alcohol (yes, no). The analyses were further adjusted for possible intermediate variables which included hypertension (yes, no, unknown), hypercholesterolemia (yes, no, unknown), diabetes mellitus (yes, no, unknown), and atrial fibrillation/atrial flutter (yes, no) diagnosed before baseline. We included continuous variables using restricted cubic splines with three knots. The assumption of proportional hazards in the Cox regressions analyses was evaluated by plotting scaled Schoenfeld residuals. 

Possible differences in the underlying dietary patterns related to intake of total fish, processed red meat, unprocessed red meat and poultry were investigated by radar charts. For these plots the intake of different foods was energy adjusted using the residual method [21]. Analyses were carried out using Stata 14 (StataCorp, College Station, TX, USA).

## 3. Results

### 3.1. Cohort and Ischemic Stroke Cases 

A total of 57,053 participants were recruited and of those 569 participants had cancer and 582 had a stroke before baseline and were excluded. Due to missing information on exposure or covariates a further 564 participants were excluded leaving 55,338 for analyses. During a median follow-up of 13.5 years, 1879 ischemic strokes occurred. When subdivided according to TOAST classifications, the cases included 319 strokes due to large artery atherosclerosis, 102 strokes due to cardioembolism, 844 strokes due to small-vessel occlusion, 98 strokes of other etiology, and 516 strokes of undetermined etiology. Baseline characteristics of the cohort and ischemic stroke cases are shown in Table 1. 

### 3.2. Total Ischemic Stroke

**Figure 1:**
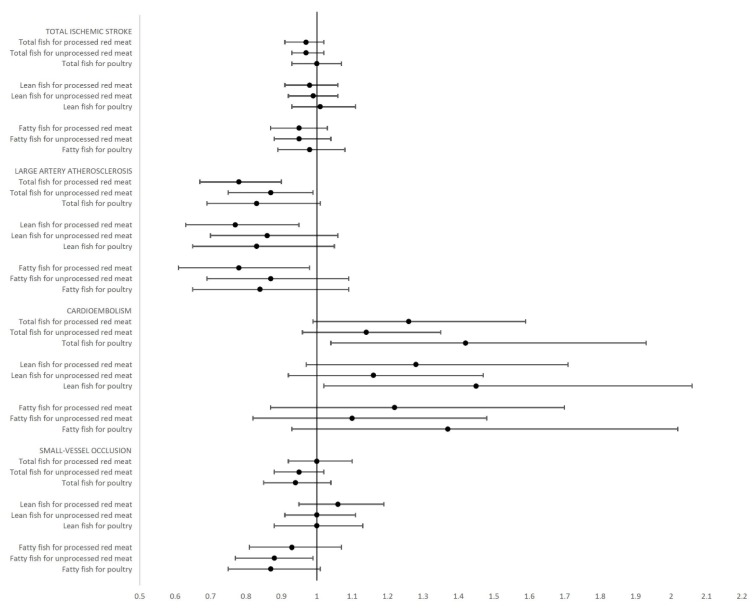
Hazard ratios and 95% confidence intervals for total ischemic stroke, large artery atherosclerosis, cardioembolism, and small-vessel occlusion when substituting total, lean, or fatty fish for processed and unprocessed red meat or poultry. Adjusted for age, sex, energy intake, length of schooling, physical activity, waist circumference adjusted for body mass index, alcohol intake, alcohol abstinence, and smoking status.

For total ischemic stroke no statistically significant associations were found when substituting total fish, lean fish or fatty fish for processed red meat, unprocessed red meat, or poultry (Table 2 and Figure 1)**.**

### 3.3. Large Artery Atherosclerosis

Regarding subtypes of ischemic stroke, statistically significant lower rates of large artery atherosclerosis were seen when total fish replaced either processed red meat (HR: 0.78; 95% CI: 0.67; 0.90) or unprocessed (HR: 0.87; 95% CI: 0.75; 0.99) red meat. When total fish replaced poultry a lower rate of large artery atherosclerosis was seen (HR: 0.83; 95% CI: 0.69; 1.01), although not statistically significant. Similar patterns of associations were seen for the same substitutions, when total fish was divided into lean and fatty fish although only statistically significant when processed red meat was replaced (Table 2 and Figure 1). In summary, for large artery atherosclerosis a lower risk was observed when red meat was replaced by fish.

### 3.4. Cardioembolism

For cardioembolic strokes a higher rate was seen, when total fish replaced processed red meat (HR: 1.26; 95% CI: 0.99; 1.59), unprocessed red meat (HR: 1.14; 95% CI: 0.96; 1.35), or poultry (HR: 1.42; 95% CI: 1.04; 1.93), although only statistically significant when total fish replaced poultry. Similar patterns of associations were found when fish was divided into lean and fatty fish, although the association was only statistically significant when lean fish replaced poultry (HR: 1.45; 95% CI: 1.02; 2.06) (Table 2 and Figure 1). In summary, for cardioembolic strokes the results indicated higher risks when fish replaced red meat or poultry intake.

### 3.5. Small-Vessel Occlusion

No clear associations were found for small-vessel occlusion when total or lean fish replaced processed red meat, unprocessed red meat or poultry. However, lower rates of small-vessel occlusion were found when fatty fish replaced processed red meat (HR: 0.93; 95% CI: 0.81; 1.07), unprocessed red meat (HR: 0.88; 95% CI: 0.77; 0.99) or poultry (HR: 0.87; 95% CI: 0.75; 1.01), although this was only statistically significant when fatty fish replaced unprocessed red meat (Table 2 and Figure 1). In summary, for small-vessel occlusion the results only indicated a lower risk when fatty fish replaced intake of red meat or poultry.

### 3.6. Further Adjustment

HRs with 95% CIs for the age-, sex-, and energy-adjusted results (Model 1A) are given in Table S1 in the Supplementary Material and showed similar patterns of associations as results from Model 1B. When analyses were further adjusted for possible intermediate variables the same patterns of association were observed (data not shown). 

### 3.7. Dietary Pattern

**Figure 2:**
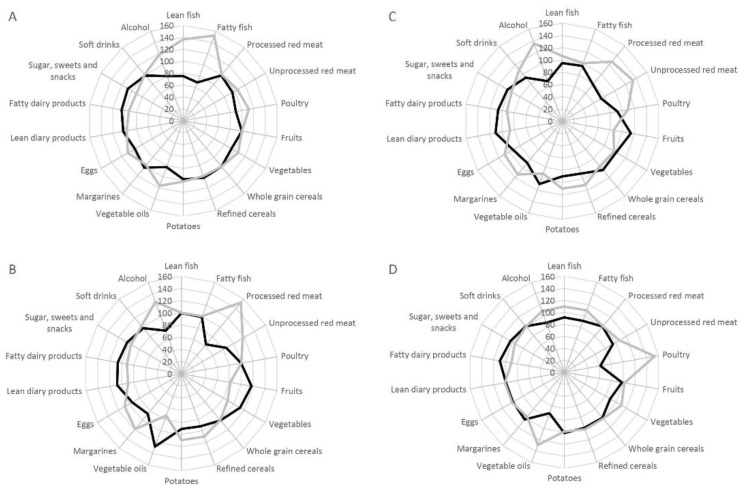
Radar charts illustrating the difference in intake of food groups and beverages according to lowest and highest intakes of (**A**) fish, (**B**) processed red meat, (**C**) unprocessed red meat, and (**D**) poultry. Highest intake is illustrated with a black line and lowest intake is illustrated with a grey line.

The underlying dietary pattern associated with high and low intakes of total fish, processed and unprocessed red meat and poultry is illustrated in Figure 2. Participants who had a high intake of total fish or poultry consumed more vegetable oils and alcohol compared with participants who had a low intake of fish or poultry (Figure 2A,D). Participants with a high intake of processed or unprocessed red meat consumed more alcohol and less fruits and vegetables oils compared with participants who had a low intake of processed or unprocessed red meat (Figure 2B,C).

## 4. Discussion

In this study, we found no association between substitution of fish for red meat or poultry and the risk of total ischemic stroke. However, we found a lower risk of strokes caused by large artery atherosclerosis when red meat was replaced by fish. A higher risk of strokes caused by cardioembolism was found when poultry was replaced by lean fish and a lower risk of small-vessel occlusion was found when fatty fish replaced unprocessed red meat.

Strengths of this study include a large number of ischemic stroke cases identified by linkage with the Danish National Patient Register independently of exposure information, which limits the risk of information bias. Furthermore, all ischemic stroke cases were validated individually and subclassified according to the TOAST classification. Also, according to a minor loss to follow-up the risk of selection bias is of little concern. Detailed information on established risk factors of ischemic stroke limits but cannot eliminate residual confounding. When we adjusted our models for established risk factors for ischemic stroke the risk estimates were weakened, indicating confounding from these risk factors. Moreover, a validated food frequency questionnaire with 24 questions was used to assess fish intake, which allowed analyses of total fish intake as well as intake of lean and fatty fish, separately. The use of statistical food substitution models allowed us to investigate and to specify food substitutions in relation to ischemic stroke risk, which may easily be translated into dietary recommendations.

Potential limitations include measurement errors related to assessment of the diet through self-reported food frequency questionnaires at baseline and potential changes in diet during follow-up are not captured. Only Danish, middle-aged participants were included in the study cohort. Hence results may not be applicable to other age groups. Also, close to 30% of total ischemic stroke cases were classified as having stroke of undetermined etiology, which includes cases with more than two potential causes identified as well as those with incomplete evaluation. Furthermore, the study participants predominantly consumed lean fish, and the results for substitution of total fish may therefore be mainly attributed to intake of lean fish. Moreover, when replacing red meat or poultry with fish, concomitant changes in other foods are likely to occur. As we did not adjust for the intake of other foods, the substitutions are thus indicators of all changes in the diet when fish intake replace intake of meat.

To our knowledge, this study is the first to investigate substitution of specified food groups in relation to ischemic stroke and its subtypes. However, Bernstein et al. [11] identified a total of 4030 strokes and found a lower risk of stroke when red meat was replaced by fish. Also, when poultry replaced fish a lower risk of stroke was found. However, this study cannot be directly compared to our study since the authors did not separate ischemic from hemorrhagic strokes. Other studies have investigated intake of fish and mainly found inverse associations with ischemic stroke risk [2,3,4]. However, most studies of ischemic stroke in relation to fish intake have compared individuals with different intake of fish but equal intake of total energy. When analyses are adjusted for total energy intake, a substitution model is introduced, although not specified. In these models, a higher intake of one food must be accompanied by a concomitant lower intake of other foods assuming energy balance [5]. Since the food items being replaced can attenuate or augment the association between fish intake and ischemic stroke risk, specifying the substitution is important. In our study, we included the sum of fish, red meat, and poultry and also each food group separately except for the food group to be replaced, hence we could estimate the rate of ischemic stroke associated with the substitution.

The associations between substitutions of fish for other foods might be mediated by several mechanisms. Fish is a source of important nutrients such as vitamin D, selenium, and iodine, and is the primary source of marine n-3 PUFA [22]. Marine n-3 PUFA has been associated with beneficial effects on various ischemic stroke risk factors including blood pressure, plasma triglycerides and inflammatory measures [6,7,8]. However, fish may also contain different pollutants e.g., methylmercury and polychlorinated biphenyls (PCBs) [23,24]. Exposure to methylmercury has been associated with progression of carotid atherosclerosis and exposure to PCBs has been associated with increased blood pressure and development of diabetes mellitus, which are risk factors for ischemic stroke [25,26]. Yet, evidence from epidemiologic studies suggests that the benefits of fish consumptions outweigh the potential harm [7,8].

While fish is rich in marine n-3 PUFA, red meat contains saturated fat, which has been associated with higher serum low-density lipoprotein (LDL) cholesterol [27]. Furthermore, red meat contains heme iron, which may be associated with oxidative stress reactions [28]. Both high LDL cholesterol and oxidative stress reactions are risk factors of ischemic stroke [29,30]. Our results suggest that the type of red meat to be replaced may also be relevant to ischemic stroke risk. Sodium is often added to processed red meat in the manufacturing process [31] and is positively associated with hypertension, which is an important risk factor for ischemic stroke [32]. In contrast to red meat, poultry contains less heme iron and high amounts of n-6 PUFA, which may be inversely associated with LDL cholesterol [33]. Although intake of n-6 PUFA may be beneficial in relation to ischemic stroke risk, it may induce more proinflammatory substances compared to n-3 PUFA [8]. Thus, replacing poultry with fish may be beneficial, as some of our results indicate.

Our findings suggest that substitution of fish may differ according to different subtypes of ischemic stroke. We found a lower risk of large artery atherosclerosis when total fish replaced processed and unprocessed red meat, suggesting a protective effect against atherosclerosis with these substitutions. Substitution of lean fish for poultry was associated with a higher risk of cardioembolism, which was unexpected. A plausible explanation could be that strokes due to cardioembolism mainly originate from atrial fibrillation and some studies have found a positive association between a high fish intake and atrial fibrillation [34,35]. However, only 102 cases with strokes due to cardioembolism were identified and interpretation of the results on cardioembolism should be done with caution. We found a lower risk of small-vessel occlusion when unprocessed red meat was replaced by fatty fish, but not when replaced by lean fish. Differences in the content of marine n-3 PUFA between lean and fatty fish might explain these results.

## 5. Conclusions

Replacing red meat with fish was associated with a lower risk of strokes caused by large artery atherosclerosis and when red meat was replaced with fatty fish it was associated with a lower risk of small-vessel occlusion, whereas replacing poultry with lean fish was associated with a higher risk of cardioembolism.

## Figures and Tables

**Table 1:** Baseline characteristics of the cohort and ischemic stroke cases.

	Cohort (*n* = 55,338)		Ischemic Stroke Cases (*n* = 1879)	
Age at enrolment (year)	56.1	(50.7–64.2)	58.8	(51.0–64.7)
Sex % (*n*)				
Male	47.6	(26,351)	61.7	(1160)
Female	52.4	(28,987)	38.3	(719)
Total energy intake (kcal/week)	15,911	(9859–24,600)	16,269	(9978–25,485)
Length of schooling % (*n*)				
<7 years	32.8	(18,177)	40.7	(764)
8–10 years	46.1	(25,515)	42.5	(799)
>10 years	21.1	(11,646)	16.8	(316)
Physical activity (hours/week)	2.5	(0.0–11.0)	2.0	(0.0–11.0)
Body mass index (kg/m^2^)	25.5	(20.5–33.3)	26.2	(20.7–34.8)
Waist circumference (cm)	88.8	(69.0–110.0)	93.0	(71.0–115.0)
Alcohol intake (g/day)	12.9	(0.7–64.6)	14.6	(0.4–79.4)
Alcohol abstain % (*n*)				
Yes	2.3	(1271)	3.2	(60)
No	97.7	(54,067)	96.8	(1819)
Smoking status % (*n*)				
Non-current smoker	64.1	(35,462)	49.6	(933)
Current smoker <15 g/day	13.0	(7214)	15.5	(291)
Current smoker ≥15 g/day	22.9	(12,662)	34.9	(655)
Hypercholesterolemia % (*n*)				
Yes	7.3	(4065)	10.5	(198)
No	50.3	(27,834)	48.9	(918)
Unknown	42.4	(23,444)	40.6	(763)
Hypertension % (*n*)				
Yes	16.0	(8865)	28.4	(533)
No	70.9	(39,226)	58.3	(1096)
Unknown	13.1	(7247)	13.3	(250)
Diabetes mellitus % (*n*)				
Yes	2.0	(1116)	4.6	(87)
No	93.4	(51,660)	89.4	(1679)
Unknown	4.6	(2562)	6.0	(113)
Atrial fibrillation/atrial flutter % (*n*)				
Yes	0.8	(423)	1.5	(28)
No	99.2	(54,915)	98.5	(1851)
Dietary intake				
Total fish	38.1	(11.3–91.5)	39.2	(11.5–94.6)
Lean fish	22.5	(6.6–57.2)	23.4	(5.7–60.2)
Fatty fish	13.5	(2.1–43.4)	13.6	(2.0–45.4)
Processed red meat	24.6	(5.2–75.1)	28.2	(6.5–84.7)
Unprocessed red meat	74.4	(29.6–158.9)	82.0	(32.6–168.4)
Poultry	17.9	(3.5–59.1)	17.2	(3.4–62.1)

Values are expressed as medians (5th–95th percentile) unless otherwise indicated.

**Table 2:** The association between substitution of fish for processed red meat, unprocessed red meat or poultry and total ischemic stroke and ischemic stroke subtypes.

	Total Ischemic Stroke	Large Artery Atherosclerosis	Cardioembolism	Small-vessel Occlusion	Stroke of Other Etiology	Stroke of Undetermined Etiology
Substitutions 150 g/week	HR (95% CI)	HR (95% CI)	HR (95% CI)	HR (95% CI)	HR (95% CI)	HR (95% CI)
Total fish						
Processed red meat	0.97 (0.91; 1.02)	0.78 (0.67; 0.90)	1.26 (0.99; 1.59)	1.00 (0.92; 1.10)	0.97 (0.77; 1.22)	0.96 (0.87; 1.06)
Unprocessed red meat	0.97 (0.93; 1.02)	0.87 (0.75; 0.99)	1.14 (0.96; 1.35)	0.95 (0.88; 1.02)	1.02 (0.83; 1.26)	1.01 (0.93; 1.11)
Poultry	1.00 (0.93; 1.07)	0.83 (0.69; 1.01)	1.42 (1.04; 1.93)	0.94 (0.85; 1.04)	1.11 (0.81; 1.54)	1.09 (0.95; 1.25)
Lean fish						
Processed red meat	0.98 (0.91; 1.06)	0.77 (0.63; 0.95)	1.28 (0.97; 1.71)	1.06 (0.95; 1.19)	0.99 (0.73; 1.36)	0.92 (0.80; 1.05)
Unprocessed red meat	0.99 (0.92; 1.06)	0.86 (0.70; 1.06)	1.16 (0.92; 1.47)	1.00 (0.91; 1.11)	1.05 (0.78; 1.42)	0.96 (0.84; 1.10)
Poultry	1.01 (0.93; 1.11)	0.83 (0.65; 1.05)	1.45 (1.02; 2.06)	1.00 (0.88; 1.13)	1.14 (0.78; 1.69)	1.03 (0.87; 1.22)
Fatty fish						
Processed red meat	0.95 (0.87; 1.03)	0.78 (0.61; 0.98)	1.22 (0.87; 1.70)	0.93 (0.81; 1.07)	0.93 (0.63; 1.36)	1.03 (0.88; 1.20)
Unprocessed red meat	0.95 (0.88; 1.04)	0.87 (0.69; 1.09)	1.10 (0.82; 1.48)	0.88 (0.77; 0.99)	0.98 (0.68; 1.41)	1.08 (0.93; 1.25)
Poultry	0.98 (0.89; 1.08)	0.84 (0.65; 1.09)	1.37 (0.93; 2.02)	0.87 (0.75; 1.01)	1.07 (0.69; 1.66)	1.16 (0.97; 1.39)

Hazard ratios (HR) with 95% confidence intervals (CI). Adjusted for age, sex, energy intake, length of schooling, physical activity, waist circumference adjusted for body mass index, alcohol intake, alcohol abstinence, and smoking status (Model 1B).

## Supplementary Materials

The following are available online at http://www.mdpi.com/2072-6643/10/11/1648/s1, Table S1: The association between substitution of fish for processed red meat, unprocessed red meat or poultry and ischemic stroke and subtypes, adjusted for age, sex, and energy intake (Model 1A).
