# Supplementary material for: Substitution of Fish for Red Meat or Poultry and Risk of Ischemic Stroke
Source: Nutrients. 2018 Nov 3;10(11):1648. doi: 10.3390/nu10111648 (PMC6266775; doi:10.3390/nu10111648)
Supplement: Supplementary file 1 [file nutrients-10-01648-s001.pdf]

Table S1. The association between substitution of fish for processed red meat, unprocessed red meat or poultry and ischemic stroke and subtypes.

	Total ischemic stroke	Large artery atherosclerosis	Cardioembolism	Small-vessel occlusion	Stroke of other etiology	Stroke of undetermined etiology
Substitutions 150 g/week	HR (95% CI)	HR (95% CI)	HR (95% CI)	HR (95% CI)	HR (95% CI)	HR (95% CI)
Total fish						
Processed red meat	0.91 (0.86; 0.96)	0.73 (0.63; 0.83)	1.23 (0.98; 1.55)	0.94 (0.86; 1.02)	0.89 (0.71; 1.11)	0.92 (0.83; 1.01)
Unprocessed red meat	0.96 (0.91; 1.01)	0.85 (0.74; 0.97)	1.14 (0.97; 1.35)	0.93 (0.87; 1.01)	1.00 (0.81; 1.25)	1.00 (0.92; 1.10)
Poultry	1.02 (0.95; 1.10)	0.84 (0.70; 1.02)	1.49 (1.09; 2.03)	0.97 (0.87; 1.07)	1.14 (0.81; 1.60)	1.12 (0.97; 1.29)
Lean fish						
Processed red meat	0.93 (0.87; 1.01)	0.74 (0.60; 0.90)	1.26 (0.95; 1.66)	1.01 (0.90; 1.13)	0.94 (0.70; 1.29)	0.88 (0.77; 1.01)
Unprocessed red meat	0.99 (0.92; 1.07)	0.86 (0.70; 1.05)	1.16 (0.92; 1.46)	1.01 (0.91; 1.12)	1.07 (0.79; 1.46)	0.96 (0.84; 1.11)
Poultry	1.06 (0.97; 1.16)	0.85 (0.66; 1.09)	1.51 (1.07; 2.15)	1.04 (0.92; 1.19)	1.22 (0.81; 1.82)	1.07 (0.90; 1.28)
Fatty fish						
Processed red meat	0.87 (0.80; 0.95)	0.72 (0.57; 0.90)	1.20 (0.87; 1.67)	0.85 (0.74; 0.97)	0.81 (0.55; 1.18)	0.96 (0.83; 1.12)
Unprocessed red meat	0.92 (0.85; 1.00)	0.84 (0.67; 1.05)	1.11 (0.83; 1.49)	0.85 (0.75; 0.96)	0.91 (0.63; 1.33)	1.05 (0.91; 1.22)
Poultry	0.99 (0.89; 1.09)	0.84 (0.64; 1.09)	1.45 (0.98; 2.14)	0.88 (0.76; 1.02)	1.04 (0.66; 1.65)	1.17 (0.98; 1.41)

Hazard ratios (HR) with 95% confidence intervals (CI). Adjusted for age, sex and energy intake (Model 1A).
